# The effectiveness and cost-effectiveness of the peer-delivered Thinking Healthy Programme for perinatal depression in Pakistan and India: the SHARE study protocol for randomised controlled trials

**DOI:** 10.1186/s13063-015-1063-9

**Published:** 2015-11-25

**Authors:** Siham Sikander, Anisha Lazarus, Omer Bangash, Daniela C. Fuhr, Benedict Weobong, Revathi N. Krishna, Ikhlaq Ahmad, Helen A. Weiss, LeShawndra Price, Atif Rahman, Vikram Patel

**Affiliations:** Human Development Research Foundation, Islamabad, Pakistan; Sangath, , Goa, India; Faculty of Epidemiology and Population Health, London School of Hygiene and Tropical Medicine, London, UK; Department of Health and Human Services, National Institute of Mental Health, National Institutes of Health, Bethesda, Maryland, USA; Institute of Psychology, Health and Society, University of Liverpool, Liverpool, UK; Centre for Chronic Conditions and Injuries, Public Health Foundation of India, Delhi NCR, India

**Keywords:** Thinking Healthy Programme, Psychological treatment, Peers, Non-mental health professionals, Perinatal depression, Task-shifting, Randomised trials, Low and middle income countries

## Abstract

**Background:**

Rates of perinatal depression (antenatal and postnatal depression) in South Asia are among the highest in the world. The delivery of effective psychological treatments for perinatal depression through existing health systems is a challenge due to a lack of human resources.

This paper reports on a trial protocol that aims to evaluate the effectiveness and cost-effectiveness of the Thinking Healthy Programme delivered by peers (Thinking Healthy Programme Peer-delivered; THPP), for women with moderate to severe perinatal depression in rural and urban settings in Pakistan and India.

**Methods/Design:**

THPP is evaluated with two randomised controlled trials: a cluster trial in Rawalpindi, Pakistan, and an individually randomised trial in Goa, India. Trial participants are pregnant women who are registered with the lady health workers in the study area in Pakistan and pregnant women attending outpatient antenatal clinics in India. They will be screened using the patient health questionnaire-9 (PHQ-9) for depression symptoms and will be eligible if their PHQ-9 is equal to or greater than 10 (PHQ-9 ≥ 10). The sample size will be 560 and 280 women in Pakistan and India, respectively. Women in the intervention arm (THPP) will be offered ten individual and four group sessions (Pakistan) or 6–14 individual sessions (India) delivered by a peer (defined as a mother from the same community who is trained and supervised in delivering the intervention). Women in the control arm (enhanced usual care) will receive health care as usual, enhanced by providing the gynaecologist or primary-health facilities with adapted WHO mhGAP guidelines for depression treatment, and providing the woman with her diagnosis and information on how to seek help for herself. The primary outcomes are remission and severity of depression symptoms at the 6-month postnatal follow-up. Secondary outcomes include remission and severity of depression symptoms at the 3-month postnatal follow-up, functional disability, perceived social support, breastfeeding rates, infant height and weight, and costs of health care at the 3- and 6-month postnatal follow-ups. The primary analysis will be intention-to-treat.

**Discussion:**

The trials have the potential to strengthen the evidence on the effectiveness and cost-effectiveness of an evidence-based psychological treatment recommended by the World Health Organisation and delivered by peers for perinatal depression. The trials have the unique opportunity to overcome the shortage of human resources in global mental health and may advance our understanding about the use of peers who work in partnership with the existing health systems in low-resource settings.

**Trial registration:**

Pakistan Trial: ClinicalTrials.gov Identifier: NCT02111915 (9 April 2014)

India Trial: ClinicalTrials.gov Identifier: NCT02104232 (1 April 2014)

## Background

Depression is one of the leading burdens of disease globally and there is a large treatment gap, especially in low- and middle-income countries (LMICs) [1, 2]. In South Asia, the prevalence of common mental disorders is reported to be among the highest in the world [3–6] ranging from 18 and 30 % in urban areas [4, 7, 8] to 28–36 % in rural areas [9–11]. In addition to constituting a burden to women’s health, disability, functioning and mortality, particularly through suicide [12, 13], perinatal depression is associated with negative child outcomes including infant under-nutrition and stunting, and has been declared a global threat to child health and development [14–16]. Perinatal depression is defined as having a depressive episode either during pregnancy or during the one-year postnatal period [17, 18].

Systematic reviews in high-income countries provide robust evidence that perinatal depression can be managed effectively with psychological treatments [19, 20]. A recent systematic review for LMICs showed the burden of perinatal depression could be reduced through mental health interventions delivered by non-specialists [21]. Apart from reducing perinatal depression, these psychological treatments have shown to improve child health outcomes like increased vaccination coverage and reduced diarrheal episodes. Such interventions have been shown to improve child health outcomes [22]. However, integrating and scaling up such interventions within the existing heath systems with them delivered by community health workers (CHWs) in LMICs is challenging because CHWs are already overburdened and are a finite human resource, and there is limited evidence of the cost-effectiveness of such interventions [2, 23, 24].

The South Asian Hub for Advocacy, Research and Education for mental health (SHARE) was established by a grant funded by the US National Institute of Mental Health (NIMH) to address these challenges. SHARE aims to adapt an established evidence-based intervention, the Thinking Healthy Programme (THP) originally developed and evaluated in Pakistan [22, 25]. THP has recently been adopted by the World Health Organisation (WHO) for global implementation (http://www.who.int/mental_health/maternal-child/thinking_healthy/en/) [26], and has now been adapted for delivery by peers (called the Thinking Healthy Programme Peer-Delivered or THPP) in India and Pakistan, through extensive formative research [27]. The peers who deliver the intervention are women with children living in the same community as the participants, and who work in partnership with established CHWs. In this paper, we describe the protocol for two concurrent randomised controlled trials being conducted in India and Pakistan, which aim to evaluate the effectiveness and cost-effectiveness of the THPP on maternal and child outcomes. Both trials have identical interventions and outcomes with some differences, which are highlighted by referring to them as SHARE Pakistan and SHARE India.

### Objectives and hypothesis

The objectives of the trials are to evaluate the effectiveness and cost-effectiveness of THPP for women with moderate to severe perinatal depression in Rawalpindi, Pakistan, and Goa, India. The primary hypothesis is that the THPP intervention provided together with enhanced usual care (EUC) will be superior to EUC alone in increasing the proportion with remission from perinatal depression and reducing perinatal depressive symptoms at the 6-month postnatal follow-up. We will test secondary hypotheses that the THPP intervention is associated with a range of maternal and child outcomes (Tables 1 and 2).

**Table 1:** Primary and secondary outcomes of the trials

	Source of data (see Table 2 for details of each measure)	
Outcomes	Measure	End point
Remission at 6 months (PHQ scores <10 and < 5) & Depression severity (PHQ scores)	Patient health questionnaire (PHQ-9)	3- and 6-month postnatal follow-up
Disability scores	WHO disability assessment schedule (WHO-DAS)	3- and 6-month postnatal follow-up
Perceived social support of the mother	Multidimensional scale of perceived social support	3- and 6-month postnatal follow-up
Rates of exclusive breastfeeding	Maternal 24-hour recall using the WHO definition of exclusive breastfeeding	3- and 6-month postnatal follow-up
Infant nutritional status	Digital scales for weight and tape measure for height (height mats)	3- and 6-month postnatal follow-up
Costs of illness	Client service receipt inventory	3- and 6-month postnatal follow-up
Serious adverse events (SAEs)	SAE form	3- and 6-month postnatal follow-up

**Table 2:** Pakistan and India trial outcome assessments

Instrument	Description	Outcome	Contextual validity
PHQ-9	Nine-item questionnaire assessment of depressive symptoms assessed on a scale of 0 to 3	Prevalence of moderate–severe depression; mean total score	Validated in primary care [29]
WHO disability assessment schedule	12-item questionnaire for measuring functional impairment over the last 30 days. In addition, two items assess the number of days the person was unable to work in these 30 days	Total disability score; quality adjusted life years; number of days out of work	Validated for international use [36]
Client service receipt inventory	Questionnaire to collect data on the utilisation and costs of health care and lost productivity (including that of caregivers)	Costs of illness (direct and indirect)	Previously used in the study settings, an adapted version will be used based on an international study on perinatal depression [37]
Multidimensional scale of perceived social support	12-item questionnaire to measure perceived social support of the mother	Perceived social support of the mother; total score	Used in the study settings [22]
Exclusive breastfeeding	24-hour maternal recall using the WHO definition of exclusive breastfeeding	Proportion of infants exclusively breastfed	Used in the study settings [38, 39]
Height and weight of infant	Digital scales for weight and tape measure for height (height mats)	Height in centimetres and weight in grams	Extensively used in the study settings [22, 40]
Serious adverse events (SAEs)	Specifically designed questionnaire for the trials	Proportion of SAEs	Specifically designed for the trials

## Methods/design

### Trial settings

The trials will be conducted in rural and urban settings (the rural sub-district of Kallar Syedan, Rawalpindi, Pakistan, and peri-urban Goa, India, respectively). Table 3 provides information about the SHARE Pakistan and SHARE India study settings.

**Table 3:** Study settings

Study settings	Pakistan	India
Study area	Kallar Syedan, one of the seven rural sub-districts of the district Rawalpindi	Three out of six sub-districts of North Goa district: Bardez, Bicholim and Tiswadi talukas.
Population	170,000	1,460,000
Average household size	6.2 members	4.2 members
Livelihood	Mainly subsistence farming, supported by serving in the armed forces or working as government employees and semi-skilled or unskilled labourers in the cities	Mainly tourism supported by mining and agriculture
Literacy rates	Males 80 %, females 50 %	Males 93 %, females 82 %
Infant mortality rates	84 per 1000 live births	9 per 1000 live births

### Design

#### SHARE Pakistan

In Pakistan, the trial will be a stratified cluster randomised controlled trial, with 40 village clusters allocated in a 1:1 ratio to the intervention and control arms. A village cluster, with a population of 2400–3600 with two or three adjacent catchment areas for lady health workers (LHWs), will form the unit of randomisation, and participants will be recruited from the community. The reason for choosing a village cluster as the unit of randomisation was to minimise contamination between trial participants.

#### SHARE India

In India, the trial will be an individual randomised controlled trial in which women attending the outpatient antenatal clinics from North Goa District Hospital and Goa Medical College Hospital will be allocated in a 1:1 ratio to the intervention and control arms. Since women attending the two hospitals come from selected areas across North Goa, the chances of contamination are less, allowing for individual randomisation. Randomisation will be stratified as rural or urban, based on the individual’s area of residence.

### Participants and procedures

**Fig. 1:**
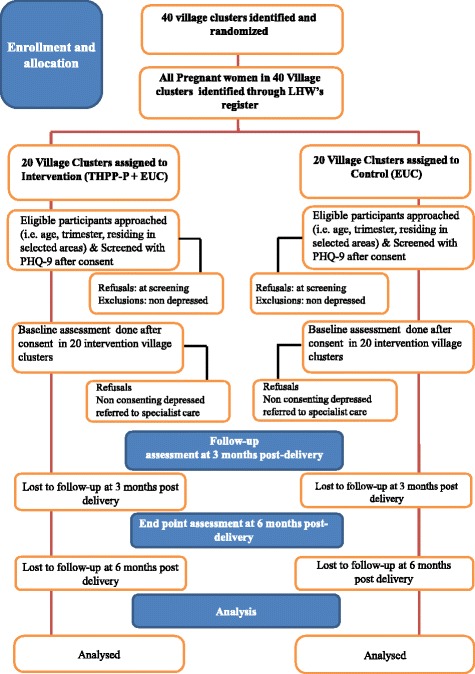
Flow chart: SHARE Pakistan trial

**Fig. 2:**
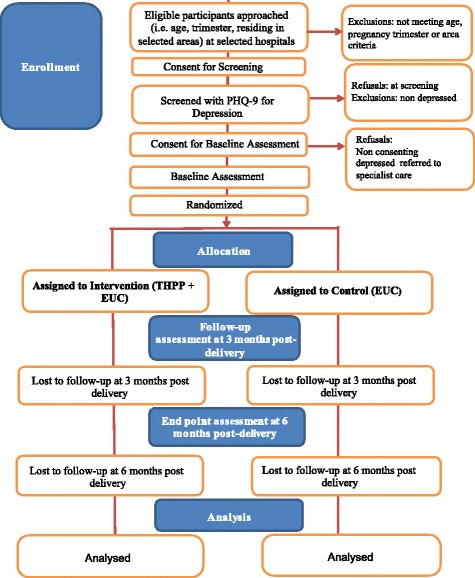
Flow chart: SHARE India trial

The trial flow charts (Figs. 1 and 2) show the process of recruitment and follow-up of participants in both trials.

#### Identification of eligible women

In Pakistan, each village cluster is covered by two or three government-employed CHWs called LHWs. The primary role of the LHWs is to provide health education and basic maternal and child health care through monthly home visits. Each LHW is responsible for about 150 households and keeps a register of every new pregnancy in her catchment area. All pregnant women living in the study area who are on the registers of the LHWs, will be checked for eligibility. The SHARE Pakistan trial has obtained a letter of permission signed by the District Health Department (Province of Punjab, Pakistan), under which the health system functions in all the village clusters, to access the LHW registers. In India, all pregnant women registering at the outpatient antenatal clinics of the hospitals will be checked for eligibility.

All potentially eligible women identified from the registers will be invited by trained research assessment teams to be screened for depression using the patient health questionnaire (PHQ-9). The questionnaire scores the nine symptom-based criteria for depression in the *Diagnostic and Statistical Manual of Mental Disorders, fourth edition* (DSM-IV) on a four-point Likert scale from not having the symptom at all, to having it nearly every day, over the last 2 weeks. The score for each item is summed to arrive at a total score. The cut-off point of 10 is selected as the most accurate value for the detection of depression [28] and has a high positive predictive value for the diagnosis of depressive disorder [29]. The PHQ-9 has been translated into local languages and previously used in both Pakistan and India [30, 31]. The original THP trial used diagnostic interviews for depression [22] and was found to be effective in reducing it. In the current trials, we choose to use PHQ-9 with a cut-off of ≥10 (i.e. moderate depression), which has good predictive value, as mentioned above.

### Inclusion criteria

Eligible participants will be pregnant women in their second or third trimester, aged 18 years and above, who intend to stay in the study area for at least 1 year and score ≥10 on the PHQ-9.

### Exclusion criteria

Women requiring immediate inpatient care for any reason (medical or psychiatric) or who do not speak Urdu, Punjabi or Potohari (Pakistan), or Konkani, Hindi or Marathi (India) will be excluded.

### Informed consent

Both trials will obtain informed written consent at screening and baseline, followed by re-affirmation of consent at the 3- and 6-month postnatal follow-ups. Informed written consent will be obtained by trained research teams, who will ensure it is taken appropriately. A copy of the information sheets and consent forms will be left with the participants.

A record of age, depression score and reasons for refusal will be maintained for those who do not consent. Separate consent will be taken, by trained research teams, for participation in the qualitative sub-study and for the audio recording of intervention sessions to monitor therapy quality. Refusals at all stages will be documented.

### Baseline assessments

Baseline assessment will entail the following information: (i) age in years, (ii) marital status, (iii) obstetric history, (iv) educational attainment, (v) employment status, (vi) treatment expectation, (vii) perceived social support and (vii) incidence of domestic violence in the last 3 months.

In Pakistan, the baseline assessments will take place at a venue of the participants’ choice –either at her own home, the LHWs’ house called Health House, or at any other household in the community. In India, the baseline assessments will take place at the hospital from which the trial participants were recruited and, immediately after they will be asked to give informed consent.

### Power calculations

The power calculations for both trials are given in Table 4 and the assumptions are given below.

**Table 4:** Power calculations: SHARE trials

Assumptions	Pakistan	India
Unit of randomisation	Villages (stratified within union councils)	Individual (stratified by urban/rural area of residence)
Intra-cluster correlation	0.07 in the intervention arm	0.05 in the intervention arm (30 therapists; within-therapist clustering) [22, 32, 33]
	0.05 in the control arm (between-village clustering) [22]	
Loss to follow-up over 6 months	20 % [22]	15 % [8]
Allocation ratio (intervention/control)	1:1	1:1
Number of participants	560 (14 per village; 40 village clusters)	280
Outcome	Power	
Remission at 6 months (PHQ-9 < 10) of 65 % in the intervention arm vs 45 % in the control arm [22]	90 %	86 %
Remission at 6 months (PHQ-9 ≤ 5) of 38 % in the intervention arm vs 22 % in the control arm [22]	81 %	74 %
Depression severity at 6 months (PHQ-9 score) – effect size 0.4	90 %	84 %

#### SHARE Pakistan

The power calculations assume the following:

1. That 40 village clusters are randomised with a 1:1 allocation ratio, stratified within 11 union councils (i.e. a stratified cluster randomised trial).
2. Intra-cluster correlation (ICC) of 0.07 in the intervention arm, and 0.05 in the control arm, to allow for between-village cluster correlation. This is a conservative estimate based on the Rahman et al. THP trial [22] in a similar population, where ICC of 0.04–0.09 was observed between union councils (which were the cluster units). We would expect a substantially lower ICC between villages within a union council.
3. That 14 participants are recruited per village cluster, with loss to follow-up of 20 % over 6 months (conservative, based on the Rahman et al. trial, which had 10 % loss to follow-up of mothers at 6 months) [22].

The sample size of 560 participants recruited provides 90 % power to detect a difference in remission at 6 months of 65 % in the intervention arm and 45 % in the control arm. This sample size also provides 90 % power to detect an effect size of 0.4 for the continuous outcomes.

#### SHARE India

The power calculations assume the following:

1. There are 25–40 peers in the intervention arm.
2. ICC of 0.05 is based on the Rahman et al. THP trial. This may be conservative as the CoBalT trial found within-therapist clustering of only 0.0027 [32] and MANAS had 0.02 [33]. We allowed for within-therapist clustering and variations in experience, competence and delivery of the intervention by the therapist. Patients randomised to one therapist may have outcomes more similar to each other than patients randomised to a different therapist. We based the estimate of the within-therapist ICC on previous studies (CoBalT trial and MANAS).
3. Loss to follow-up of 15 % over 6 months (based on the Goa postnatal depression cohort study, which reported an attrition rate of 13 % over 6 months) [8].
4. Equally sized groups (1:1 allocation ratio) due to the relatively small ICC.
5. Remission rates at 6 months of 45 % in the control arm and 65 % in the intervention arm (based on the Rahman et al. THP trial, which had recovery rates of 47 % and 77 % respectively; slightly lower recovery rates are assumed as the intervention is being delivered by peers).

### Randomisation

#### SHARE Pakistan

Within each of the 11 union councils (of the sub-district), village clusters will be randomised to the intervention and control arms using a 1:1 allocation ratio. Randomisation will be done before the participants are recruited. Research teams responsible for identifying, obtaining consent and recruiting trial participants will be blind to the allocation status. This will help to minimise the post-randomisation recruitment bias. Stratification by union councils will minimise the imbalance between arms by factors likely to be associated with the primary outcome and reduce the between-cluster variability, hence increasing the power of the study. Allocation of clusters will be carried out by an independent statistician based at the London School of Hygiene and Tropical Medicine (LSHTM) using a computerised randomisation sequence.

#### SHARE India

Treatment allocation of participants will be carried out after informed consent is obtained and the eligible women have completed their baseline assessments and seen the doctor for their antenatal check-up. Stratification by urban or rural place of residence will minimise imbalance between arms, since pilot data indicated differences in prevalence of depression between rural and urban areas, hence increasing the power of the study. Allocation will be carried out using sequentially numbered opaque sealed envelopes [34] prepared by an independent statistician at LSHTM.

### Interventions

#### Thinking Healthy Programme Peer-delivered

THP has been adopted by WHO for global implementation as part of the mhGAP series on low-intensity psychological interventions [26]. The original THP was developed and tested in Pakistan where it was delivered by government-employed LHWs [22, 25]. THP was adapted for peer delivery by undertaking an extensive formative phase prior to setting up the trials. In total, 61 in-depth interviews (IDIs) and 3 focus group discussions were conducted in Goa, and 38 IDIs and 10 focus group discussions in Rawalpindi [27]. The formative research findings along with the process findings from the original trial provided the rationale for adapting and further simplifying the key strategies for delivery by peers. In THPP, the focus is on identifying the mother’s unhealthy behaviours (rather than cognitions as in the original THP), replacing them with healthy behaviours, and practising these (focus on behavioural activation).

In Pakistan, the peers are called *razakaar* (meaning ‘volunteer’), while in India they are called *sakhis* (meaning ‘friend’). In Pakistan, the peers will be working in partnership with the community-based LHWs, who run women’s groups. The intervention starts from the third trimester of pregnancy until the sixth postnatal month and includes ten home-based individual face-to-face sessions and four group-based sessions (given in the LHW women’s groups). Part of the task sharing by peers is to assist LHWs in organising and conducting their existing routine women’s groups and to encourage trial participants to attend these sessions. These group sessions (within the intervention arm only) will be held at the house of the local LHW (called a Health House). All these women will continue to receive their local LHW’s routine monthly care visits.

In India, the intervention sessions will start from the second or third trimester of pregnancy until the 6-month postnatal follow-up. The intervention will be delivered through individual face-to-face sessions (minimum 6 and maximum 14 sessions). The venue for session delivery, agreed collaboratively with the trial participants, will primarily be at the participants’ homes.

Peers will be lay women, without any mental health training background who have shown an interest or desire to help and support other women within their community. These peers will be from the same communities as the trial participants or nearby communities. All the peers will be married, mothers and fluent in the local languages of the trial settings in Pakistan and India. All peers will be identified and recruited from the local community through word of mouth, particularly through community-based key informants such as the LHWs in Pakistan, and women’s group facilitators and *anganwadi* workers (community health-care workers responsible for the well-being and nutrition of mothers and newborn children) in India.

The peers will be trained in a 5-day classroom-based workshop (approximately 4–5 hours daily). This will be followed by a 2-month internship where they can practise the content of the THPP on non-trial participants. During this time, they will receive regular monitoring and competency checks, and be supervised. The training format will be based on interactive discussions and role-playing and will cover the following topics: content of the THPP intervention, confidentiality, dealing with difficult situations, and detection and reporting of serious adverse events (SAEs). In Pakistan, the LHWs will be part of the training session that covers confidentiality for peers. This will help ensure confidentiality of the participants by both the peers and LHWs. This will be refreshed and reiterated in monthly supervision meetings. Peers will also receive training on how and when to communicate with the research teams if any of these situations arise, and to take the necessary steps to trigger the referral pathway and an assessment of the trial participant by an independent psychiatrist. Peers will be assessed, at the end of their training, on their competence using structured and standardised role-playing. This will indicate any further training needs. Only peers who achieve the desired level of competence will be selected to deliver THPP in the trials.

Peers will receive monthly group-based supervision by trained intervention facilitators (who will be part of the research teams). The monthly supervision will include revision of the THPP content through discussions and role-playing. The discussions will cover the challenges faced by peers in the field, issues around confidentiality, detection of SAEs, problem-solving and keeping peers motivated.

#### Enhanced usual care

Participants will receive EUC in both the intervention and control arms, as there is non-existent usual care for perinatal depression. The majority of cases of perinatal depression either remain unidentified or are classified as pregnancy blues in both settings. There is no provision of services for mothers with depression in the primary health-care system of Pakistan or in hospitals in Goa. EUC will comprise the following:

1. Informing participants about their diagnosis of depression.
2. Informing depressed participants about ways to seek appropriate health care (i.e. by going for assistance to their LHWs, to the primary health centre or to the tertiary health centre, which is the Institute of Psychiatry, Rawalpindi, Pakistan).
3. In India, providing gynaecologists with the findings of the screening results for perinatal depression.
4. Providing the primary health-care centres and the gynaecologists with the adapted WHO mhGAP treatment guidelines for perinatal depression [35].
5. Providing an information sheet to all pregnant women about how and where to seek health care from including local Community Health Workers (CHWs), primary health facilities and tertiary care facilities, both during pregnancy and beyond.

### Outcome evaluation

The primary end point of the trial is 6 months postnatal; in addition, interim follow-up data will also be collected at 3 months postnatal. At each follow-up, consent will be reaffirmed by trained research assessment teams. The outcome assessment measures with the specific outcomes are given in Tables 1 and 2, and they are described briefly below.

#### Patient health questionnaire

PHQ-9 scores the nine DSM-IV symptom-based criteria for depression on a four-point Likert scale from not having the symptom at all, to having it nearly every day, over the last 2 weeks. The score for each item is summed to arrive at a total score. The cut-off point of 10 is selected as the most accurate value for the detection of depression [28] and has a high positive predictive value for the diagnosis of depressive disorder [29]. PHQ-9 has been translated into local languages and previously used in both Pakistan and India [30, 31].

#### WHO disability assessment schedule

The WHO disability assessment schedule (WHO-DAS) is a 12-item questionnaire for measuring functional impairment over the last 30 days. In addition, two items assess the number of days the person was unable to work in the past 30 days. It gives a total disability score, quality adjusted life years and number of days out of work. WHO-DAS has been validated for international use [36].

#### Client service receipt inventory

This questionnaire collects data on the utilisation and costs of health care and lost productivity (including that of caregivers). It will provide the costs of illness (both direct and indirect). It will be used at the 3- and 6-month follow-ups to cover costs of illness starting from antenatal to 6 months postnatal. Client service receipt inventory (CSRI) has been used in the study settings, and an adapted version will be used based on an international study on perinatal depression [37].

#### Multidimensional scale of perceived social support

The multidimensional scale of perceived social support is a 12-item questionnaire that measures the perceived social support of the mother. It has three domains of support: (1) significant other, (2) family and (3) friend. It is a based on a Likert scale and the higher the score, the more the perceived social support. It has been used in the study settings [22].

#### Exclusive breastfeeding

This will be based on 24-hour maternal recall using the WHO definition of exclusive breastfeeding. It has been used in the study settings [38, 39].

#### Infant anthropometry

Digital scales for weight and a tape measure for height (height mats) will be used. Z-scores for both weight for age and height for age will be calculated. This has been used extensively in the study settings [22, 40].

### Discontinuation criterion

Trial participants will be discontinued from the study if there is:

- Loss of baby or miscarriage
- Development of a psychotic episode
- Development of a manic episode

### Minimisation of contamination

The risk and level of contamination will be monitored at the levels of the peers and participants. All peers will be instructed not to share any information about the study, and not to provide any support to other mothers in the community, other than the ones who are assigned to the peer for delivery of THPP. This procedure will be regularly reviewed at the peers’ monthly supervision meetings. For the Pakistan trial, the risk of contamination will be minimised by the cluster design. The intervention and control village clusters will be geographically separated and the chance of intervention cluster participants regularly meeting control cluster participants will be negligible. Apart from this, both trials also plan to carry out a social network analysis of participants at the 3- and 6-month postnatal follow-ups to capture if any sizeable contamination took place and to adjust accordingly in our final analysis, using a contamination-adjusted intention-to-treat (ITT) analysis. The extent of social networking will be assessed at the level of the participants so that statistical adjustments can be made through contamination-adjusted ITT analysis (complier average causal effect analysis) [41, 42].

### Masking

Due to the nature of the intervention, it is not possible to mask participants and peers to treatment allocation. However, outcome evaluation will be masked, as all outcome measures will be administered by trained research assessment teams (outcome assessors), who are non-residents of the study area, independent of the intervention and masked to treatment allocation of the participants. Furthermore, participants will be instructed not to disclose to the outcome assessors whether they are receiving the intervention or not. During all assessments, the primary outcome measure (PHQ-9) will always be completed first to minimise the risk of bias in the event of unmasking and, if it occurs, the point of unmasking will be recorded. Sensitivity analyses will be carried out to assess the effect of unmasking on the primary outcomes. The intervention and outcome assessment teams will not have any interactions during the trial since there will be separate locations and administrative management. The assessment teams will be told that they are evaluating two interventions and that there is genuine equipoise about which one is better.

### Fidelity of the intervention

Two types of indicator will be collated, in both trials, to evaluate the fidelity of the THPP intervention, namely, quantity and quality. Quantity indicators will include:

1. Number and duration of sessions delivered; information will be collated from peer logbooks, which will record basic information about the session, such as date, time and duration of the session
2. Number of participants who complete the treatment and have a planned discharge
3. Number of participants who are treatment failures or need referral to mental health specialist services for any other reason

#### SHARE Pakistan

In Pakistan, the quality of sessions delivered by peers will be assessed using a specifically designed competency checklist based on the six key areas of THPP and the ENACT scale (which is an 18-item assessment for common factors in psychological treatments), including task-sharing initiatives with non-specialists across cultural settings [43]. Peers’ level of competency will be assessed using this checklist, during trainings and monthly supervision meetings with role-playing and sessions conducted by peers in the field.

#### SHARE India

In India, the quality of sessions delivered by peers will be assessed using the therapy quality scale. This is an 18-item scale that consists of two separate subscales: treatment-specific skills, which are specific to an intervention (e.g., reviews previous session, assigns homework and involves family members), and treatment approach skills, which assess common counselling skills that the counsellor uses (e.g., using active listening, appropriate language and a collaborative approach). This scale is based on a 20-item therapy quality scale used to evaluate the delivery of a culturally adapted depression treatment (the Healthy Activity Programme) by lay health workers in primary health-care centres in Goa [44], which is known to have good psychometric properties [45]. For competency, the peers will be rated on their performance in standardised role-playing with specific checklists for each session.

All sessions will be audio recorded and 5 % of these will be purposively selected for transcription and rated for fidelity by psychological treatment experts. All peers and different sessions will be covered within the 5 %. This was not found to be acceptable to women in Pakistan during piloting of the intervention, therefore it was not proposed for the trial phase.

### Nested qualitative study

Trial participants will be purposively selected for qualitative interviews after completing the 6-month outcome assessments. The aim of the qualitative study is to explore the trial participants’ perceptions of the intervention, the quality of the care received, their satisfaction with the peer, and the impact of their health problems on themselves and their children. We expect 40 women to be approached for IDIs, which will continue until data saturation is reached. These interviews will include trial participants from both intervention and control arms, women who withdraw from the trial, participants who recover and those who remain depressed at the final end-point assessment. Apart from the trial participants, we will also conduct IDIs with peers and health system personnel to take their views and perceptions about the linkage and task-sharing aspects of the intervention. All interviews will be audio recorded and transcribed. Analysis will be done manually using a thematic framework approach. The qualitative team will be separate to the intervention and outcome assessment teams.

### Data management

Baseline and follow-up outcome data will be captured electronically using tablet computers. In addition, process data from peers will be collected. Baseline and outcome data will be remotely uploaded as a CSV (comma-separated values) file to the main data server, which will run the software programs STAR and ODK in India and Pakistan, respectively. Both files will be compliant with good clinical practices (including a date and time stamp of original data entry and with an audit trail to document any subsequent changes). Process data will be collected in paper form, and this will be manually entered and stored as CSV files using the same data collection platform. THPP quality-related data will also be collected in paper form. All data, range and consistency checks will be performed at weekly intervals separately for each data source. Any queries identified will be resolved promptly by the data management team, and the database will be updated, maintaining the audit trail. All types of data will be kept in separate databases and only merged into a master database after data collection is completed and each individual database is locked. Access to the data will be protected by password at multiple levels and no member of the trial team apart from the data managers will have access to these passwords. The database will be backed up daily, with off-site storage on external hard drives and DVD media.

Qualitative data will be collected using digital recorders together with written field notes and memos. The former will be transcribed in the language of the interview, anonymised but linked with the trial ID and translated for analysis. A similar procedure will be followed for the written data. Digital recordings will be stored in a secure, password-protected folder.

### Analysis

The quantitative analysis will be done using STATA (version 13) following CONSORT guidelines for individually randomised and cluster-randomised trials respectively. Flow charts will include the number of participants seen at each stage of the trial, including the number screened, eligible, randomised and analysed for the primary outcome. Initial analyses will compare baseline characteristics of enrolled participants by arm and follow-up status. The outcome measures will be summarised at baseline and the 3- and 6-month postnatal follow-ups by intervention arm, summarised by means (standard deviation), medians (interquartile range) or numbers and proportions as appropriate (and including age, gender and baseline outcome score). For continuous outcomes, histograms within each arm will be plotted to assess how closely the scales follow a normal distribution to determine how to describe the outcomes and how to do the inferential analysis properly.

The primary analyses will be ITT at the 6-month follow-up visit adjusted for the baseline measure of the outcome. In the Pakistan trial, mixed-effects logistic regression models will be used to analyse binary outcomes, adjusting for village clusters as random-effects variables and Union Councils as fixed effects. In the Indian trial, peers will be adjusted for as a random effect in the analysis. Analyses of continuous outcomes will use mixed-effects linear regression, additionally adjusting for the baseline value of the outcome. Adjustments will also be made for variables for which randomisation did not achieve a balance between the two arms at baseline.

Effect sizes will be reported as: (1) crude and adjusted relative risks estimated using the marginal standardisation technique with 95 % confidence intervals for the ratios estimated via the delta method [46] for binary outcomes, (2) and as mean differences and standardised mean differences, with 95 % confidence intervals for continuous outcomes. Missing data for the outcomes will be estimated using multiple imputation [47]. Secondary analyses will include repeated measures analyses of data collected at the 3- and 6-month postnatal follow-ups, which is efficient use of all available data.

#### Economic analysis

Service utilisation and the out-of-pocket expenditures of the participants and the infants (costs for seeing a doctor or other health-care provider, admission to hospital, medicines, tests and extra help at home) will be collected in both trials (at the 3- and 6-month postnatal follow-ups). The data collected through the CSRI will be used to calculate service costs and total costs of care for each participant. Unit costs of services itemised in the CSRI – such as cost per outpatient visit – will be based on locally conducted health facility costing exercises. Service cost data will subsequently be linked to primary and secondary study outcomes, in particular depression and disability summary scores (PHQ-9 and WHO-DAS, respectively), to assess issues around the value or cost-effectiveness of the task-shifting intervention. In the event that dominance is not shown, i.e. the THPP intervention is more effective but the costs are also more than in the EUC group, incremental cost-effectiveness ratios will be computed, together with their confidence intervals (using bootstrapping techniques to overcome the expected skewness of the cost data). Results will be plotted on a cost-effectiveness plane and presented as cost-effectiveness acceptability curves to show the probability of the intervention being cost-effective at a range of willingness-to-pay threshold levels. A sensitivity analysis will be conducted to take account of uncertainty and imprecision in the measurements, including multiple imputation models for missing values.

#### Compliance analysis

A proportion of participants, in both trials, may comply poorly with the THPP intervention. A complier average causal effect analysis will be undertaken, which estimates the effect of the intervention on the participants who received it as intended by the original randomisation. This assumes that participants in the EUC arm have the same probability of non-compliance as do participants in the THPP arm. We will also adjust the analysis for contamination between arms (i.e. if participants in the EUC arm were exposed to the intervention through contact with intervention arm participants).

#### Moderator analysis

Moderator analyses will be used in both trials to help clarify on whom and under what circumstances (moderators) the THPP intervention works. We will assess modification of treatment effect by a priori defined modifiers (age group, socio-economic status, education, parity, chronicity of depression, baseline severity of depression and patient expectations), by fitting appropriate interaction terms and testing for heterogeneity of treatment effects in regression models. We will also carry out mediator analysis to see what key behaviours our intervention activated or affected that led to improved outcomes at 3 and 6 months. The hypothesised mediators include behavioural activation (assessed by the premium abbreviated activation scale), perceived social support (assessed by the multidimensional scale of perceived social support) and mother–child attachment (assessed by items of the maternal postnatal attachment scale).

### Trial management

The progress of the trials will be monitored by three committees. Table 5 shows the role and scope of these committees. Trial monitoring and auditing will comprise the collation and reporting of routine trial process indicators and SAEs. Summary statistics and graphs showing trends over time will be compiled for the process indicators, and reported weekly to the trial management committee (TMC), monthly to the trial steering committee (TSC) and twice per year to the data safety and monitoring board (DSMB) in the USA.

**Table 5:** Trial management committees

Committee	Role	Members	Frequency of meeting
Trial management committee (TMC)	Monitor all aspects of the conduct and progress of the trial, ensure that the protocol is adhered to, in particular the protocol related to the monitoring and reporting of SAEs, and take appropriate action to safeguard participants and the quality of the trial itself.	Principal investigators	Weekly
		Site principal investigators	
		Trial managers	
		Research team leads	
		Intervention team leads	
		Data managers	
Trial steering committee (TSC)	Provide overall governance and oversight of the trial and ensure that it is being conducted in accordance with the protocol and the relevant regulations. The TSC must agree the final trial protocol and any protocol amendments, and provide advice to the TMC on all aspects of the trial. Decisions about termination of the trial or substantial amendments to the protocol are the final responsibility of the TSC. The TSC comprises the sponsors and funders, NIMH being the funder and LSHTM and University of Liverpool being the sponsors of SHARE India and Pakistan, respectively. Both sponsoring institutions will be responsible for individual sites.	NIMH staff	Monthly
		Trial statistician and Data Coordinating Centre (LSHTM, UK) staff	
		Members of the TMC	
Data safety and monitoring board (DSMB)	The DSMB has been set up by NIMH, USA, to work specifically with the Global Hubs trials. The DSMB will ensure the safety of participants. The board will review and approve the study protocols, informed consent and all relevant documents and procedures. It is responsible for site monitoring and audit of the progress of the study, including recruitment and retention of participants, adverse events, SAEs and adherence to the time line of the study, and for making recommendations about the continuation, modification or termination of the study, based on the balance of adverse events and beneficial outcomes. Apart from this, the sites will submit reports twice per year with details of data collection and adverse events.	NIMH staff	Six monthly

### Ethical considerations

The trial protocols have been granted ethical approval from the respective sites, the Institutional Review Board of Human Development Research Foundation (Pakistan), Institutional Review Board of Sangath (Goa, India), Indian Council of Medical Research, Observational/Interventions Research Ethics Committee of LSHTM, UK, and the Committee of Research Ethics at University of Liverpool, UK. Lastly, both trial protocols have ethical approval granted by the Global Mental Health DSMB of NIMH, USA.

Written (or witnessed or audio recorded, if the participant is illiterate) informed consent will be mandatory for enrolment. Separate consent will be taken for participation in the qualitative study. All participants will be able to access EUC, which represents a higher quality of care than what is currently available at both sites. We will protect the confidentiality of personal data principally through procedures to separate study data and participant-identifiable data. Quantitative data gathered in the tablets for each participant at baseline will retain personal identification items to minimise errors in transcribing identities, but these will be removed before transferring the data to STATA for analysis. We will monitor the occurrence of a number of specific SAEs, which include death of the participant due to any cause, suicide attempt, violence against the participant, stillbirth, miscarriage or loss of child, stigmatisation (due to the intervention), any violence towards others, in particular the participant’s children, hospital admission due to a psychiatric problem and hospital admission of the participant or infant due to a serious medical emergency. Their detection, appropriate response (involving an independent psychiatrist responding within 3 working days, if requiring immediate response) and reporting will be governed by the standard operating procedure approved by the DSMB, which sets out the a priori criteria for unblinding of adverse events (i.e. a statistically significant difference in prevalence between the two arms). SAEs will be compiled by the data manager and a blinded summary report shared with the principal investigators, local ethics committees and the DSMB.

## Discussion

The SHARE THPP trials will provide evidence on the effectiveness and cost-effectiveness of an empirically supported and WHO-mandated psychological treatment delivered by peers in an LMIC context.

There is a compelling need to address maternal mental health [48], which is linked to poor child development [16], increased risk of comorbidity and maternal mortality, and the existing treatment gap. The SHARE trials have the potential to address all of the above, that is, (i) burden of perinatal depression, (ii) child outcomes linked with perinatal depression and (iii) reducing the treatment gap.

The trials, set in two very different contexts (rural and urban), are designed to establish a partnership of an untapped human resource (in the form of peers) alongside existing health systems, have a structured training and supervision model for peers, employ minimum exclusion criteria for participation and include a comprehensive economic assessment. This in turn will help the external validity of the trial findings and provide a potential model for scaling up in similar resource-poor settings of LMICs as well as for well-resourced contexts (where a mental health treatment gap exists). Overall, the trials provide a unique opportunity for global lessons on the mechanisms of identifying, recruiting, training and supervising lay peers, working in partnership with the existing health systems in two different settings.

The main limitation of our trials is that we are not using diagnostic interviews for perinatal depression. However, if the results are scaled up, it will be highly unlikely that trained mental health professionals would be diagnosing depression at the primary care level, especially in resource-poor settings.

## Trial status

Both the Pakistan and India trials began recruitment of participants in October 2014. The Pakistan trial, based on our previous work and pilot results with an approximately 25 % rate of perinatal depression, expects to recruit the sample within 12–15 months (by January 2016). The end-point assessments of all the participants at the 6-month postnatal follow-up will be completed by the end of September 2016. The Indian trial, based on our previous work and pilot results with an approximately 8 % rate of perinatal depression, is expected to recruit the sample within 19 months (by April 2016). The final outcome assessments of all the participants at the 6-month postnatal follow-up will be completed by end of April 2017.

## Abbreviations

CHW: community health worker
CSRI: client services receipt inventory
CoBalT: Cognitive Behavioural Therapy trial conducted in UK
CONSORT: Consolidated Standards of Reporting Trials
CSV: Comma separated values
DSM-IV: *Diagnostic and Statistical Manual of Mental Disorders, fourth edition*
DSMB: data safety and monitoring board
ENACT Rating Scale: Enhancing Assessment of Common Therapeutic factors rating scale
EUC: enhanced usual care
IDI: in-depth interview
ICC: intra-cluster correlation coefficient
ITT: intention-to-treat
LHW: lady health worker
LMIC: low- or middle-income country
LSHTM: London School of Hygiene and Tropical Medicine
MANAS: Effectiveness trial using lay counsellors for depression and anxiety in Goa, India
mhGAP: Mental health gap treatment guide
NIMH: National Institute of Mental Health
ODK: Open data kit, which is a software platform for tablets
PHQ: Patient Health Questionnaire
SAE: serious adverse event
SHARE: South Asian Hub for Advocacy, Research and Education in mental health
THP: Thinking Healthy Programme
THPP: Thinking Healthy Programme – Peer Delivered
TMC: trial management committee
TSC: trial steering committee
WHO: World Health Organisation
WHO-DAS: World Health Organisation disability assessment schedule
